# On the Treatment of Dorso-Lumbar Abscess by Means of Free Incisions and Subsequent Carbolized Injections

**Published:** 1882-10

**Authors:** Norman H. Chapman

**Affiliations:** Late of the Philadelphia Orthopœdic Hospital and Infirmary for Nervous Diseases, and the Pennsylvania Hospital, etc.


### ON THE TREATMENT OF DORSO-LUMBAR ABSCESS BY MEANS OF A FREE INCISION AND SUBSEQUENT CARBOLIZED INJECTIONS. By NORMAN H. CHAPMAN, M. D. M. S., late of the Philadelphia Orthopœdic Hospital and Infirmary for Nervous Diseases, and the Pennsylvania Hospital, etc.

On February 7th, 1882, Mark C., æt. 55 years, married, a painter, whose weight when taken sick was 208 lbs., came under my observation with the following history:

Twelve weeks ago was taken sick after an exposure to cold and damp, with a chill, fever of low type, pain in right side and general malaise confining him to bed. The aid of several local physicians was from time to time summoned, with the result of giving slight temporary relief, but the progress of the case was invariably downward, never gaining at any time sufficient strength to leave the bed. His true condition was never taken cognizance of or at all events was never made known to him, nor was anything radical ever suggested for his relief.

Upon examining the case I found a large strumous or cold abscess occupying the dorso-lumbar region upon the right side, which upon being carefully mapped out gave evidence of containing somewhat more than a pint of fluid. The man's general condition from long confinement in bed, and from the constant drain of the formation of pus, was very poor. He had lost over one hundred pounds in weight, and was indeed in almost the last stage of exhaustion from inanition, and the drain of suppuration. He was exceedingly feeble, very restless, delirious at times, and unable to obtain sleep without a heavy opiate. Hectic fever with afternoon exacerbations and evening declines had set in, the variations in temperature ranging from 2° to 3.5° F. The countenance when not flushed with hectic fever was pale and sallow, and the facial expression was that of a great anxiety. The patient did not suggest much pain, and there were none of the evidences of acute inflammation about the tumor, but his generally enfeebled condition was so extreme that I could not consider it safe to subject him to the necessary shock of an operation.

In the treatment of this case I therefore at first instituted a process of over-feeding, and of mild stimulation, by giving the stomach all it could very well handle of such simple and easily digested articles of diet as milk, milk punch, animal broths, gruels, oysters and the like, together with Wyeth's preparations of beef, wine and cinchona. As this systematic over-feeding, a veritable process of stuffing, was kept up from day to day with such slight variations as were most pleasing to the palate, a gradual improvement in the man's general condition was noticed. At the expiration of ten days the hectic fever had abated, he was no longer delirious, did not require opiates and wore a more cheerful expression of countenance.

In consequence of the improvement in the man's general condition I now considered it safe to operate, and was preparing to do so when word came that as the result of an accident which occurred while the patient was sleeping, the abscess ruptured. The family being much alarmed hastily bound up the parts with compress and bandage so that at the time probably not much more than one-third the contents of the cavity escaped. Upon removing the cloth some five or six hours after the rupture took place, I enlarged the opening by incision to about three-quarters of an inch, in order to give free vent to the fluid, and at this time took from the cavity upwards of fourteen ounces of thick scrofulous pus before it was completely emptied of its contents. When the cyst was well emptied an ordinary eyed probe could be entered into the cavity its entire length in almost any direction, and swept about from side to side, with perfect freedom.

At the first dressing of the abscess it was well distended and thoroughly washed out by syringe, with a one-twentieth aqueous solution of carbolic acid. And in order to protect the parts against the farther ingress of air as much as possible, the opening was carefully covered with the carbolic oil (1 part of carbolic acid to 15 of olive oil) and lint dressing. Over this a pad of oakum was placed, and a large compress so adjusted to the parts as to produce considerable pressure upon the walls of the collapsed abscess, the whole being held in place by a roller. Each day this method of treatment was continued, being careful each time the dressings were changed and the cavity syringed out, that the wound or opening was constantly enveloped in a strong vapor of carbolic acid.

For forty-eight hours after the first dressing the secretion of pus was very rapid, from three to four ounces somewhat decomposed, being removed at each daily dressing. After this time the discharges assumed a more healthy character, and the quantity grew rapidly less. The manner of syringing out and dressing the parts, already mentioned, was practiced every day until the latter part of February, when about two-thirds of the cavity was found to be obliterated. From this time on the carbolic injections were repeated with less frequency, *i. e.* at intervals of two or three days, as the character of the discharge would seem to indicate, and upon several occasions the carbolized solutions used were as strong as one to eight.

Under this treatment the size of the abscess continued steadily to diminish, the man's general health and appearance to improve, and on March 14th, he got up, and four days later his weight with overcoat was 108 pounds. Upon March 20th, the cavity was almost entirely obliterated, the probe only entering to a depth of about three-quartes of an inch. At this time the man's general condition was so much improved that he resumed work. In the course of the fortnight following, and under the stimulation of lunar caustic, the remainder of the cavity healed from the bottom by granulation and recovery was then complete.

I also desire to mention that this patient had years before been the subject of primary syphilis, and that during his recent illness there were evident some of the tertiary manifestations of the disease, but he was not, however, placed upon specific constitutional treatment until he was up and about. He was then given liberally of mercury and the iodides.

May 15th. At this date, two months after the patient first got up, he is in excellent health, is rapidly gaining in strength, and in flesh; weight now 168 pounds, and it is constantly increasing. The patient thinks that in a few months more he will entirely regain his old weight and vigor. As to the condition of the side there are no physical evidences whatever of returning suppurative trouble.

*Remarks.*—In the treatment of this case it was my intention to free the abscess from pus at the earliest possible moment, not by means of the valvular incision of Abernethy, or aspiration, but by a free incision under the carbolic acid spray, after the manner of Lister, and subsequently to follow up the treatment systematically distending and washing out the cavity with moderately strong carbolized solutions. Moreover, the man's general health when I first saw him was in such a precarious condition that any such decided interference with the parts involved must needs prove fatal. I therefore felt called upon to employ every means at my command to support and build up the general health, carefully husbanding every gain which the patient made, and meanwhile, to wait patiently in anticipation of the time when the powers of the patient should be deemed sufficient to enable him to bear up under the necessary shock of an operation. When I had considered this time at hand, however, spontaneous evacuation intervened, rendering an operation unnecessary, and fortunately the accompanying shock very light.

There is a point here in reference to the entrance of air into a pus cavity which should not be overlooked. It is one of the first principles of Lister's treatment as applied to the management of chronic abscess "that during the whole treatment the opening into the abscess shall never for a single second, be exposed to air unmixed with a strong vapor of some volatile antiseptic."* In this case, however, atmospheric air unimpregnated with any antiseptic both came in contact with the opening, and entered the cavity of the abscess, without materially interfering with the subsequent efficiency of the carbolized drainage. The cavity of a scrofulous or cold abscess is lined with a more or less well developed pyogenic membrane, and if after its evacuation it be well distended with a moderately strong carbolized solution, whether air may have entered or not, the character of the lining membrane is changed by the cauterent effect of the acid, and the destruction of atmospheric impurities secured. I do not mean to imply that atmospheric air may freely whistle in and out of the cavity of a cold abscess without doing injury, for such would be the merest absurdity, but the fact which I wish to insist upon is that should air chance to gain access to such a cavity, by accident or otherwise, the occurrence is not an event which need be the cause of undue alarm, because its injurious effects may be at once counteracted by distending the cyst with a moderately strong antiseptic solution. The solution used should be strong enough to change the nature of the lining membrane of the cavity, and to impregnate all of its discharges. Besides this the cyst should be regularly washed out with the carbolized solutions at such intervals as will prevent the discharges from decomposing, and every attention paid to the external dressings of the part.

I have employed aspiration in cases similar to the one related above, but the operation has invariably required to be several times repeated, and the results have never been altogether satisfactory. The line of treatment which would appear to be the most promising of a good result in the management of a cold abscess, and particularly in such as are located in the dorso-lumbar

*Erichsen's Science and Art of Surgery, new edition, vol. i., p. 137.

region, as I have in a measure illustrated by the history of Mark C's case, is that by a free incision into the most dependent portion of the sac under the vapor of a strong antiseptic; and afterwards by the forcible distention of the suppurating cavity with an antiseptic solution—preferably carbolic acid.

By this mode of procedure is gained a complete evacuation of the cavity, permanent relief of tension, free drainage and no decomposition of discharges. Resolution will in this way be secured, not by the uncertain absorption of pus as after aspiration, nor by granulation, but by contraction of the cavity, together with the approximation and agglutination of its walls.—*Vaughn's Diamond, Kansas City, Mo.*

---

THE NEW MEDICAL AND DENTAL DIRECTORY for Chicago and the suburbs, which has been gotten up by Mr. C. A. Kendrick, now of this city but formerly of Cincinnati, is completed, and was issued during this month (September). Besides the names, addresses and telephone facilities of the physicians and dentists, there is a very valuable list of professional nurses, whose names have been furnished Mr. Kendrick by leading physicians of the city. The chart also contains a list of the medical publications, medical and dental societies, surgical and dental depots, veterinary surgeons (graduates) and a full list of the medical institutes of the city, such as colleges, dispensaries, hospitals, infirmaries, etc.

Mr. Kendrick's address is No. 70 Monroe street, care of Dr. E. C. Dudley.

The cost to each subscriber is $1.50, including a chart, and some six hundred names have already been enrolled. The design of the publisher, it may be added, is to place copies of the chart in every drug store and medical institution of the city, and the principal hotels.

